# Cost-effectiveness of the recombinant zoster vaccine in the German population aged ≥60 years old

**DOI:** 10.1080/21645515.2018.1509645

**Published:** 2018-09-06

**Authors:** Desirée Van Oorschot, Anastassia Anastassopoulou, Barbara Poulsen Nautrup, Lijoy Varghese, Alfred von Krempelhuber, Mohamed Neine, Stéphane Lorenc, Desmond Curran

**Affiliations:** a GSK, Wavre, Belgium; b GSK Munich, Germany; c EAH Consulting, Aachen, Germany; d GSK, Singapore, Singapore; e Freelance, on behlaf of GSK, Wavre, Belgium

**Keywords:** herpes zoster, shingles, postherpetic neuralgia, cost-effectiveness, economic analysis, public health, older adults, vaccination

## Abstract

Each year, around 300,000 Herpes Zoster (HZ) cases are observed in the German population, resulting in costs over €182 million to society. The objective of this study was to estimate the potential public health and economic impact of the new Adjuvanted Recombinant Zoster Vaccine (RZV, *Shingrix*) in the German population ≥ 60 years of age (YOA) and to identify the optimal age of vaccination. We used a static, multi-cohort Markov model that followed a hypothetical cohort of 1 million people ≥ 60 YOA life-long after vaccination using German-specific inputs. Both costs and outcomes were discounted at 3%, the incremental cost-effectiveness ratio (ICER) was calculated based on the societal perspective. The coverage of RZV was set at 40% with a second-dose compliance of 70%. Vaccinating the population aged ≥ 60 YOA would result in 45,000 HZ cases avoided, 1,713 quality-adjusted life years (QALYs) gained at a total cost of approximately €63 million compared to 38,000 cases avoided, 1,545 QALYs gained at a total cost of approximately €68 million in the population ≥ 70 YOA. This would result in an ICER of approximately €37,000 and €44,000/QALY, for the age cohort ≥ 60 and ≥ 70 YOA, respectively. Scenario analyses demonstrated that vaccinating at age 60 or 65 YOA would show greater public health impact and would result in the lowest observed ICER compared to vaccinating at 70 YOA. In conclusion, starting vaccination with RZV in the German population ≥ 60 YOA would demonstrate the best value from a public health and economic standpoint.

**Figure:**
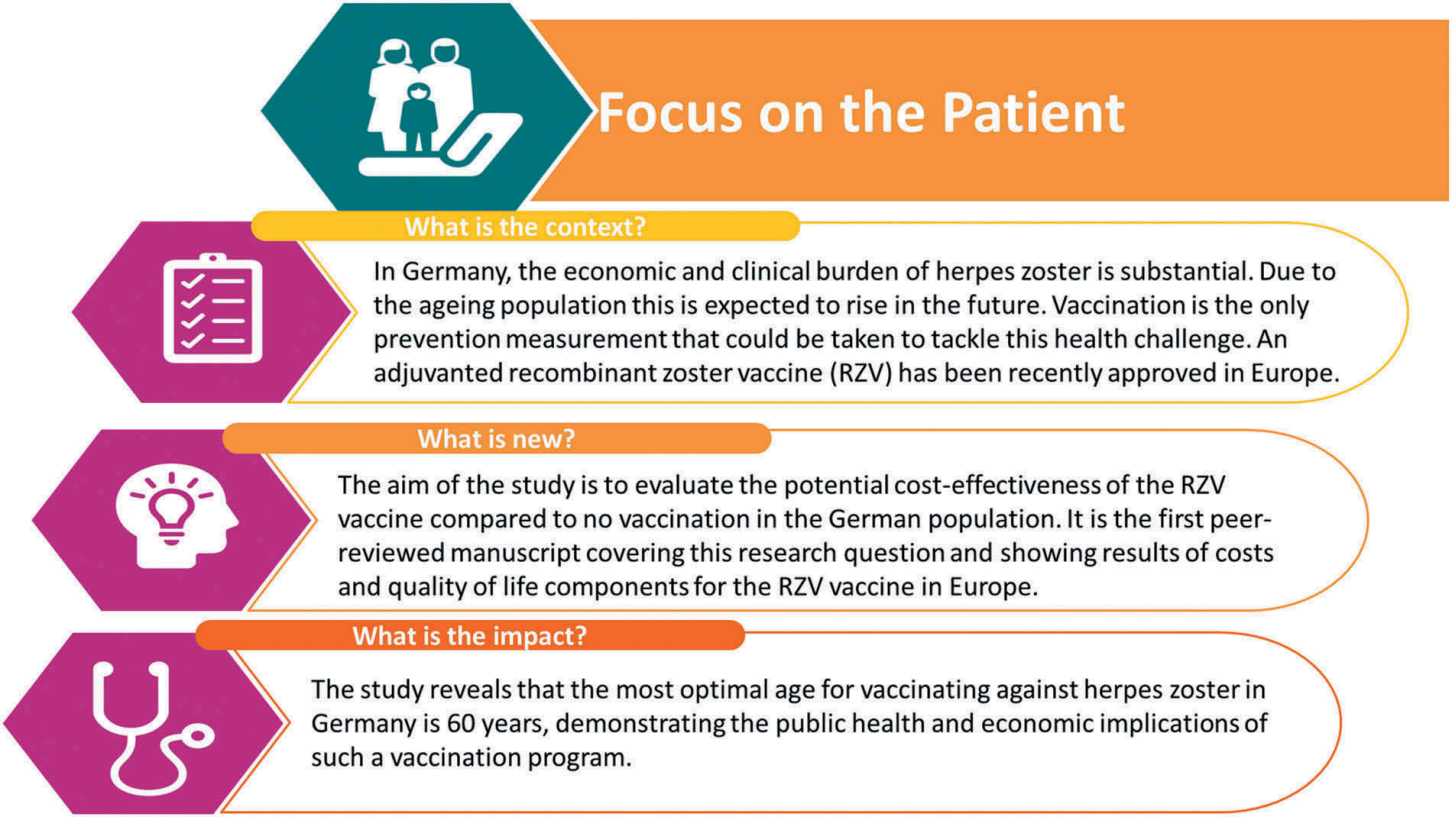

## Introduction

Before implementation of routine childhood varicella vaccination in 2004, the lifetime risk of acquiring a varicella-zoster virus (VZV) infection in Germany was more than 95%. In most cases, VZV infection occurs in children and manifests in the form of chickenpox.^1,2^ After recovery from the primary infection of VZV, the virus remains latent life-long in individual’s dorsal root ganglia.^3^ Immunosenescence or immunosuppressing illnesses and medications result in a decrease in VZV-specific T-cell immunity. This leads to reactivation of the latent VZV causing Herpes Zoster (HZ), also known as Shingles.^4,5^

HZ is usually characterized by a painful and self-limiting rash localized in the sensory region of the affected ganglia (most often the skin of the trunk or the head).^4^ The most frequent complication is postherpetic neuralgia (PHN), which is defined as pain persisting or appearing ≥ 90 days after rash onset.^6^ Other complications include ophthalmic and neurological symptoms.^6^HZ and associated complications can result in a significant reduction of patients’ quality of life.^7,8^

The burden of disease related to HZ and its complications is substantial. Each year, around 300,000 HZ cases are observed in the German population ≥ 50 years of age (YOA) including an estimated 18,000 PHN cases.^9^ The economic burden in Germany from a societal perspective is estimated to be €182 million (M) per year and is expected to rise due to changing demographics.^9^ Therefore, vaccination could help reduce the burden of disease.^10,11^

The German Standing Committee on Vaccination (STIKO) is the National Immunization Technical Advisory Group (NITAG) at the Robert Koch Institute (RKI) that makes recommendations for licensed vaccinations to be included in the routine vaccination schedule.^12,13^ One of their aims is to reduce the burden of HZ including complications and long-term consequences caused by HZ in older adults by vaccination.^14^ However, based on a systematic literature review of all available data, STIKO decided in 2017 against recommending a standard HZ vaccination with the Zoster Vaccine Live (ZVL, *Zostavax*), licensed by the European Medicines Agency (EMA) in 2006.^14,15^

A new Recombinant Zoster Vaccine (RZV, *Shingrix*), formulated with adjuvant to address age-related decline in immunity, has been licensed in 2017 in the US and Canada and recently received market authorization by the European Medicines Agency (EMA).^16^ This vaccine is administered in two doses, with the second dose recommended between 2 and 6 months after the first dose. Two large phase III clinical trials have been conducted to assess the vaccine efficacy (VE) for RZV. In people ≥ 50 YOA, the VE was 97.2%, whereas in people ≥ 70 YOA, the VE was 91.3%.^17–19^

A recent study estimated the potential public health impact of both ZVL and RZV in the total population ≥ 50 YOA in Germany. Assuming a 40% coverage for both vaccines and a second-dose compliance of 70% for RZV vaccination would lead to a reduction of 0.5M and 1.75M HZ cases when introducing ZVL or RZV, respectively.^19^ This result was achieved primarily due to the higher, sustained VE, of the RZV vaccine compared with ZVL.

Building further upon the public health impact research, the objective of this study was to evaluate the cost-effectiveness of RZV vaccination compared to ‘no vaccination’ in the population ≥ 60 YOA in the German statutory health insurance (SHI) setting. The comparison to ‘no vaccination’ was based on the current situation in Germany where ZVL is not recommended by STIKO.^14^ Exploratory analyses were also performed to assess age cohorts that could benefit the most from the vaccine in terms of public health and economic impact. Outcomes of this study could provide useful insights for a potential RZV vaccination policy in Germany.

## Results

### Base-case results

A cohort of a total of 1M subjects ≥ 60 YOA (split into age cohorts 60–64, 65–69, 70–79, ≥ 80 YOA), of which 40% were vaccinated with the first dose (at 60, 65, 70 or 80 YOA, respectively) and 70% would receive a second dose of RZV, was followed throughout the model. Over the cohort’s lifetime, approximately 45,500 HZ and 8,500 PHN cases could be avoided, which would result in a reduction of almost €14.9M and €2.8M in direct and indirect costs, respectively. Vaccination costs would be around €81M, yielding an incremental cost-effectiveness ratio (ICER) of about €37,000 per quality-adjusted life year (QALY) gained (Table 1).

**Table 1.** Base-case results; RZV vs no vaccination for the population ≥ 60 and ≥ 70 YOA.

	≥ 60 YOA	≥ 70 YOA
Population size	1M	1M
HZ Cases Avoided	45,327	37,537
PHN Cases Avoided	8,740	7,571
HZ-related Deaths Avoided	14	18
QALYs Gained (discounted)	1,713	1,545
Direct Costs prevented (discounted)	€14,898,562	€12,616,872
Indirect Costs prevented (discounted)	€2,788,907	€547,619
Vaccination Costs (discounted)*	€81,110,940	€81,101,345
ICER	€37,025/QALY	€43,969/QALY

*The small differences between the two cohorts are due to the fact that there is a risk of people dying from natural causes in between the two doses of RZV.

HZ: herpes zoster; ICER: incremental cost-effectiveness ratio; PHN: postherpetic neuralgia; QALY: quality-adjusted life year; YOA: years of age.

Vaccinating a cohort of 1M people ≥ 70 YOA (split into age cohorts 70–79 and ≥ 80 YOA), under similar coverage assumptions, would lead to a reduction of approximately 37,500 HZ and 7,500 PHN cases. This would result in €13.2M savings to society. As for the cohort ≥ 60 YOA, vaccination costs would be around €81M. The ICER from the societal perspective would be about €44,000/QALY gained, thus being less cost-effective than vaccinating the cohort ≥ 60 YOA.

### Scenario analyses

To assess the optimal age of vaccination, cohorts of 1M people 60, 65 or 70 YOA were assessed, both in terms of public health and economic impact. For all age cohorts, public health and economic impact was assessed (Table 2). The greatest number of HZ cases was avoided considering vaccination in the 60 YOA cohort, whereas slightly more PHN cases were avoided considering vaccinating the 65 YOA cohort. The number needed to vaccinate (NNV) was similar for all three age cohorts (range 7–9) but the NNV for PHN was higher (48) for the cohort 70 YOA than for other age cohorts (39). The ICERs for both 60 and 65 YOA cohorts would be similar, whereas again, this would increase for the 70 YOA cohort. The predicted costs per case prevented for HZ and PHN would be the lowest in the cohort 60 YOA, €983 and €5,477, respectively.

**Table 2.** Scenario Analyses results; RZV vs no vaccination for the population 60, 65 and 70 YOA.

	60 YOA	65 YOA	70 YOA
Population size	1M	1M	1M
HZ cases avoided	57,256	54,322	42,238
PHN cases avoided	10,274	10,398	8,480
NNV to prevent 1 HZ case	7	8	10
NNV to prevent 1 PHN case	39	39	8
QALY gained (discounted)	1,906	1,984	1,690
Direct + Indirect costs prevented (discounted)	€24,853,647	€22,622,144	€14,708,441
Vaccination costs	€81,123,991	€81,123,991	€81,091,692
***ICER***	***€29,528/QALY***	***€29,484/QALY***	***€39,282/QALY***
***Costs per HZ case avoided***	***€983***	***€1,077***	***€1,572***
***Costs per PHN case avoided***	***€5,477***	***€5,626***	***€7,828***

HZ: herpes zoster; ICER: incremental cost-effectiveness ratio; NNV: number needed to vaccinate; PHN: postherpetic neuralgia; QALY: quality-adjusted life year; YOA: years of age.

### Sensitivity analysis

#### Deterministic sensitivity analyses (DSA)

**Figure 1.**
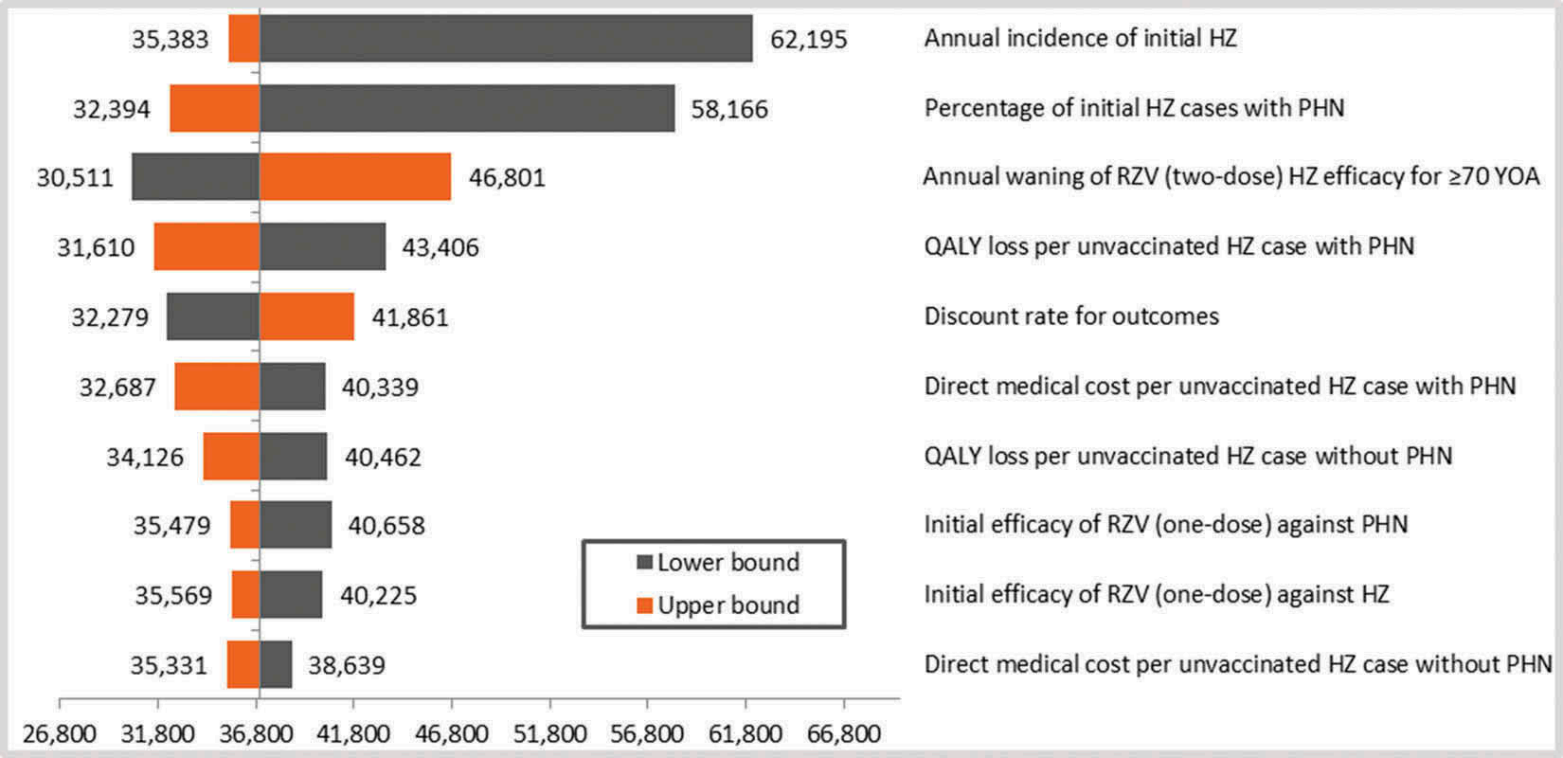
DSA top-10 results for the cohort aged ≥ 60 YOA. Base-case ICER: €37,025/QALY.DSA: deterministic sensitivity analysis; HZ: herpes zoster; PHN: postherpetic neuralgia; QALY: quality-adjusted life year; RZV: adjuvanted recombinant zoster vaccine; YOA: years of age.

**Figure 2.**
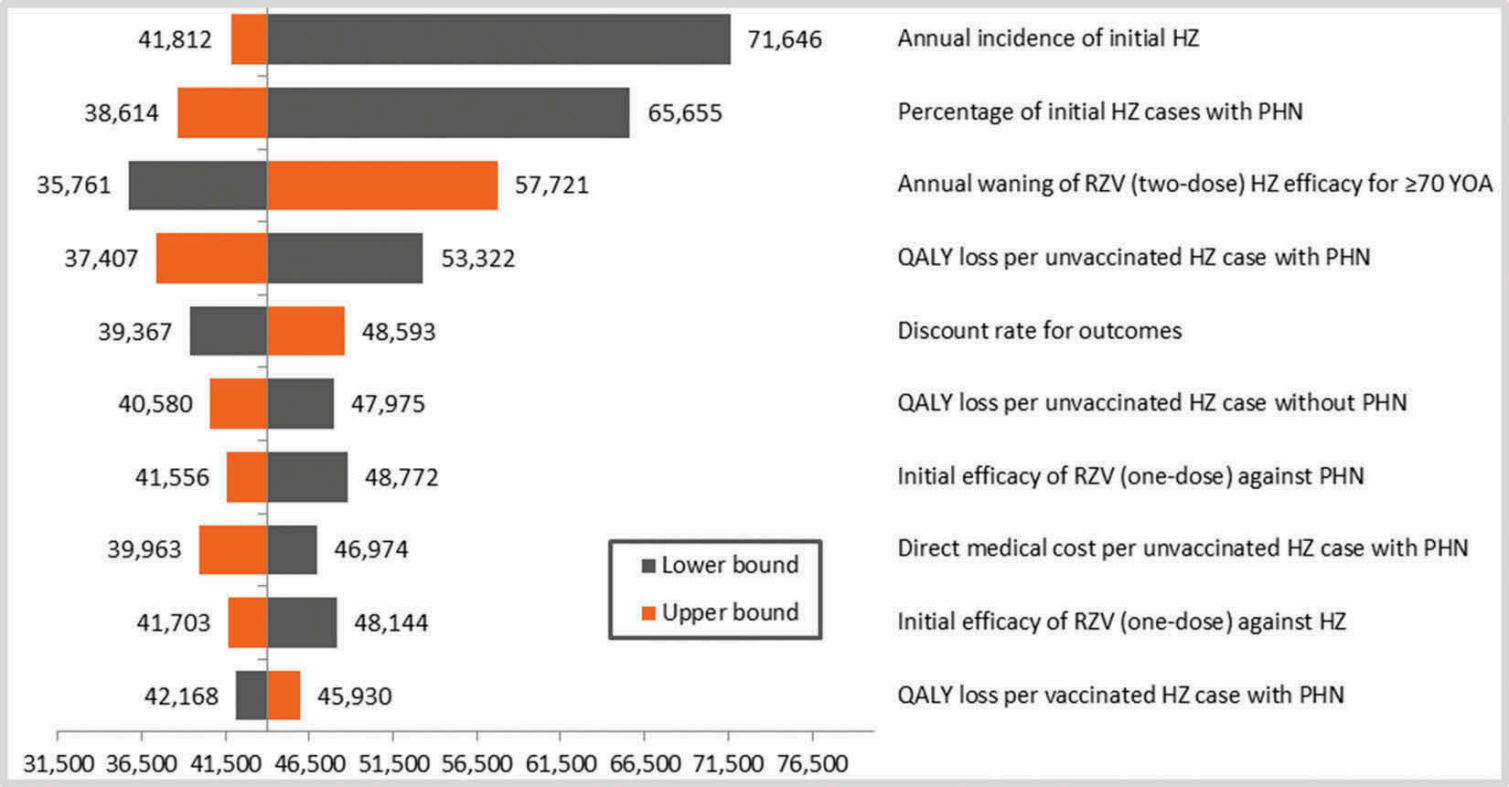
DSA top-10 results for the cohort ≥ 70 YOA. Base-case ICER: €43,969/QALY.DSA: deterministic sensitivity analysis; HZ: herpes zoster; PHN: postherpetic neuralgia; QALY: quality-adjusted life year; RZV: adjuvanted recombinant zoster vaccine: YOA: years of age.

The results of the deterministic sensitivity analyses (DSA) are given in Figure 1 (cohort ≥ 60 YOA) and Figure 2 (cohort ≥ 70 YOA). Although all parameters were varied according to the ranges provided in the input table (Table 1), only the top-10 results were presented in the tornado diagrams. Both age cohorts showed similar top-10 results, although only the first 5 parameters were in a similar order to each other. Incidence of HZ and probability of subsequently developing PHN showed the largest variation around the ICER.

#### Probabilistic sensitivity analyses (PSA)

**Figure 3.**
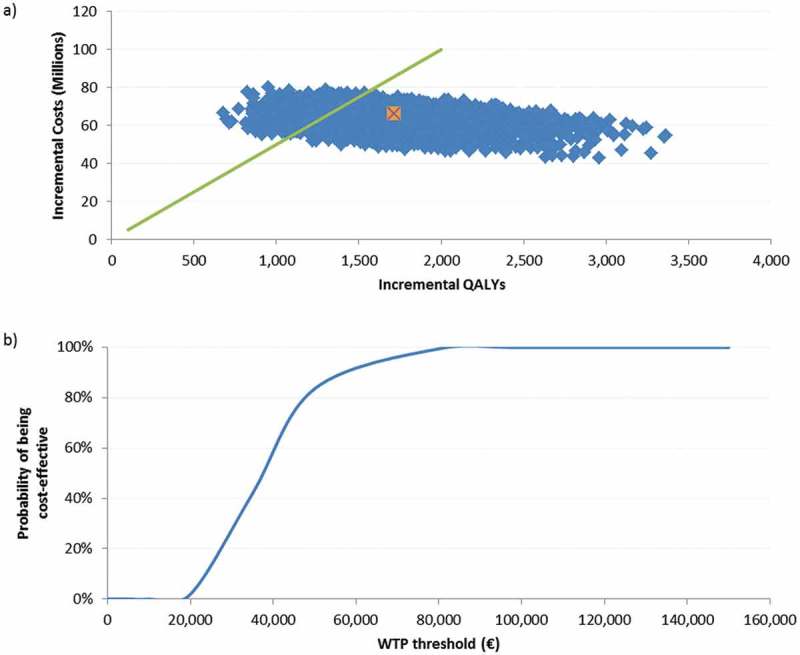
Population ≥ 60 YOA – a) PSA results for the of 5,000 Monte-Carlo simulations. b) Cost-effectiveness acceptability curve. a) The orange dot is indicating the base-case ICER of €37,025/QALY, the green line presents a hypothetical WTP threshold of €50,000/QALY. PSA: probabilistic sensitivity analysis; QALY: quality-adjusted life year; WTP: willingness to pay; YOA: years of age.

**Figure 4.**
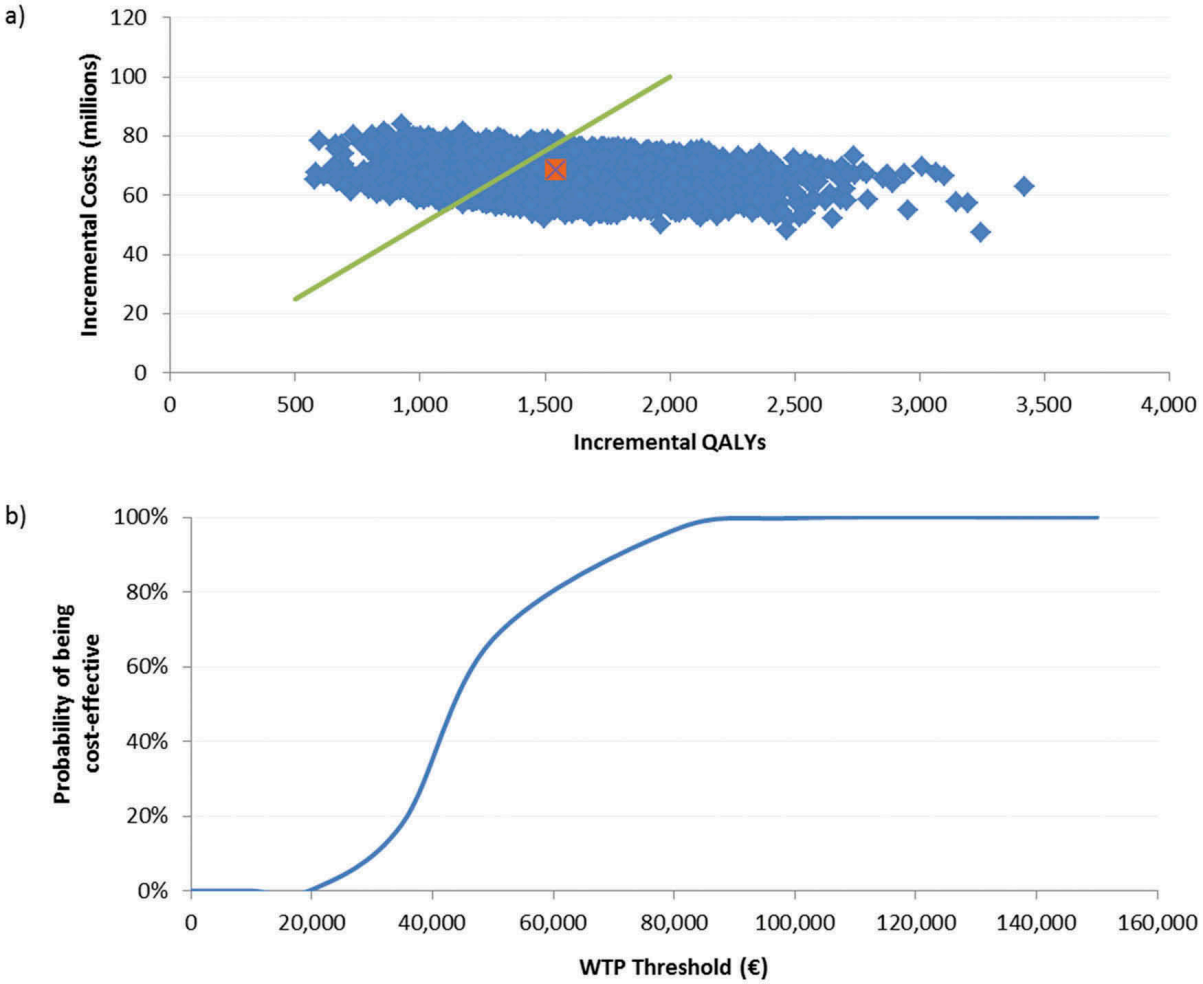
Population ≥ 70 YOA – a) PSA results for the of 5,000 Monte-Carlo simulations. b) Cost-effectiveness acceptability curve. a) The orange dot is indicating the base-case ICER of €43,969/QALY, the green line presents a hypothetical WTP threshold of €50,000/QALY. PSA: probabilistic sensitivity analysis; QALY: quality-adjusted life year; WTP: willingness to pay; YOA: years of age.

For both analyses on the cohorts ≥ 60 and ≥ 70 YOA, the probabilistic sensitivity analyses (PSA) results were shown in a scatter plot, of 5,000 Monte-Carlo simulations around the base case, given in orange. We set the willingness to pay (WTP) to a hypothetical threshold of €50,000/QALY. The cost-effectiveness acceptability curve, showing the probability of being cost-effective under various thresholds, indicated that in 84% of all simulations in the cohort ≥ 60 YOA, the ICER would be below the WTP threshold. For the population ≥ 70 YOA, 67% of all simulations would be below this threshold. (Figures 3 and 4)

**Figure 5.**
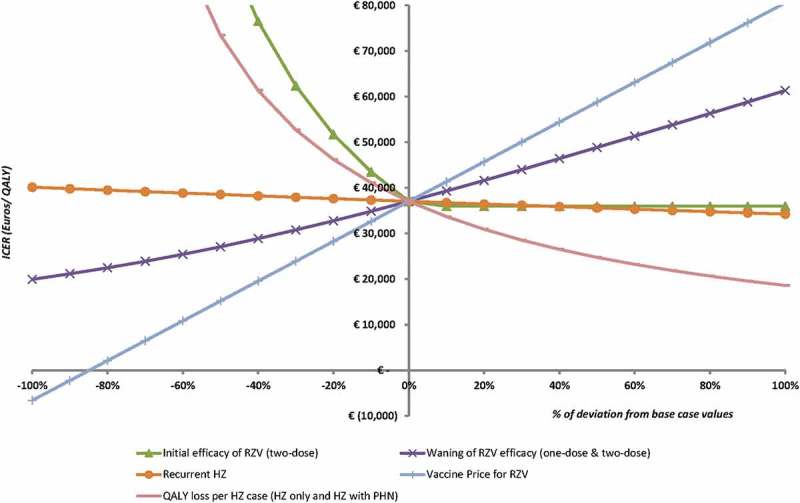
Results from the threshold analyses, indicating the change in ICER by varying the values of the parameters by steps of 10%.

#### Threshold analyses

To demonstrate the effect of uncertainty around various parameters, threshold analyses for the base case (≥ 60 YOA) were performed as presented in Figure 5. To reach the hypothetical WTP threshold of €50,000/QALY, either the price should increase by 30%, or the efficacy for two doses should decrease by approximately 20% or the waning of the 2-dose vaccine should increase by 60%. HZ recurrence has the lowest impact on the ICER, setting this value to zero results in an ICER of approximately €40,000/QALY.

#### Validation

Unlike our ZOster ecoNomic Analysis (ZONA) model, where the vaccine price for RZV was set at €110/dose (price to retailer, including obligatory discounts and rebates), the STIKO model assumed a RZV price similar to the one for the currently available ZVL (€84/dose, price to retailer). It followed patients as of 50 YOA with vaccination from 60 YOA and returned an ICER of €23,934/QALY from the societal perspective.^20^ Comparatively, while keeping all other parameters similar to the base case and only decreasing the price of RZV to €84/dose, the ZONA model showed an ICER of €26,673/QALY, from a societal perspective.

## Discussion

The objective of this study was to assess the potential cost-effectiveness of introducing RZV vaccination to the population ≥ 60 YOA in the German SHI setting. Because of the recent recommendation not to introduce ZVL by STIKO, the cost-effectiveness analysis was performed against no vaccination. To estimate the optimal age of vaccination with RZV, we demonstrated both the public health and economic implications of introducing this new vaccine. In the overall population ≥ 60 YOA, the ICER was around €37,000/QALY. This result was robust to various sensitivity analyses. The PSA showed that in 84% of all 5,000 simulations, the ICER was below a hypothetical threshold of €50,000/QALY. Increasing the age to start vaccinating everyone ≥ 70 YOA led to an ICER of approximately €44,000/QALY. It may seem counterintuitive that the predicted ICER would be higher for people ≥ 70 YOA than ≥ 60 YOA because both incidence of HZ and the probability of developing PHN increase with age. However, the prolonged duration of protection would imply that RZV contributes significantly to the benefit and this is more limited in older age groups due to the higher competing risk of death.

RZV demonstrated high efficacy in all age groups ≥ 50 YOA and clinical trials showed sustained VE up to four years in all study age groups.^17,18^ Because of these vaccine characteristics, vaccinating at a younger age seems to be more aligned to the expressed aim of the STIKO to reduce HZ disease burden in older adults.^14^ To optimize the use of RZV, scenario analyses have been performed, showing the public health and economic impact when vaccination started at 60, 65 or 70 YOA. Both 60 and 65 YOA cohorts showed an ICER of approximately €29,500/QALY, being the optimal age cohort in terms of cost-effectiveness. Vaccinating with RZV at 60 YOA, leads to a few additional HZ cases avoided for slightly lower costs compared to 65 YOA.

Extensive sensitivity analyses have been performed on most of the input values and with selected ranges based on the published literature. The DSA showed that, in both age cohorts tested, HZ incidence and probability of developing PHN were the most influential parameters. For the lower bound of HZ incidence, we referred to Schmidt-Ott et al., who presented results from a study involving physician networks, showing a relatively low HZ incidence in Germany.^21^ A systematic review by Pinchinat *et al*. reported overall HZ incidence in Europe to be higher.^4^ The lower bound of the PHN probability was derived from Horn *et al*.^22^ Other HZ cost-effectiveness models reported higher estimates on these parameters.^23,24^ Increasing the HZ incidence and PHN probability leads to a more cost-effective ICER. The ICER remained relatively stable when varying the second-dose compliance in the DSA from 50 to 90%, and thus was not presented in the top-10 results on the tornado diagram. However, it is interesting to note that the impact on public health is considerable when increasing the compliance rate, as presented in the previous publication of the ZONA model.^19^ Threshold analyses revealed that the price to patient should be increased by 30% to come close to the frequently used cost-effectiveness threshold of €50,000/QALY, whereas it also demonstrated that HZ recurrence had almost no impact on the ICER. If the QALY loss due to HZ was decreased by approximately 25% the ICER could reach the threshold. It has to be noted that this falls below all QALY values used in other cost-effectiveness analyses of HZ vaccines with the exception of Hornberger et al.^25^ In the ZONA model the estimated base case QALY loss per HZ case (including PHN) ranged from 0.040 to 0.047 in subjects aged 60–64 and ≥ 80 year olds, respectively. This is in the same range as reported by Kawai et al.,^25^ who estimated that based on the utility values reported in Ultsch et al.^10^ the QALY loss due to HZ in Germany was approximately 0.04 and 0.05 for 60–74 and ≥ 75 year olds, respectively. Larger QALY (utility) loss input values were used in the US CDC model and an independently developed model.^26,27^

One of the limitations of this study is the unknown duration of protection after 4 years, as also discussed in Curran et al. 2017.^19^ To analyze the uncertainty around the waning of efficacy, threshold analyses were performed, results demonstrated that the waning should be increased by approximately 60% to reach the WTP threshold. The data included in this model reflects the German population ≥ 60 YOA, which includes also people with an underlying condition that might have an influence on their disease pattern. Future research is therefore necessary to estimate the cost-effectiveness specifically for sub-populations such as patients with an immunocompromised condition.

The vaccination costs for a cohort of 1M people, considering the coverage of 40% and the second-dose compliance of 70% would be of approximately 81M Euros. 40% coverage is likely not to be established within the first year of the program, and therefore these costs will be spread over the years until the coverage is reached. The model follows hypothetical cohorts of 1 million people to ensure that comparisons on the outcomes can be made between age groups. If one wishes to calculate the impact on the actual size of these age groups, these numbers can be multiplied by the total number of people per age group. For example the total number of HZ cases avoided when vaccinating the 60 – 64 YOA cohort are 57,256 per 1 million people, the actual cohort size is 5,2M and thus 57,256 * 5.2 = 297,731 HZ cases could be prevented in this age cohort.

Given the fact that, to date, no other peer-reviewed study assessed the cost-effectiveness of RZV in a European setting, it is difficult to put our results into perspective. However, the STIKO conducted a comparative analysis with ZVL and RZV to inform its voting members.^20^ Assuming a parity price of for both vaccines (84€/RZV dose), they calculated an ICER of €23,934/QALY for RZV vaccination, which is close to the ICER of €26,673/QALY returned by the ZONA model, used in this analysis, when considering the same price. Both models assumed a linear waning but the STIKO model is however more conservative on this point as it used the waning data from the ZOE-70 clinical trial for all subjects instead of using ZOE-50 data for people 50–69 YOA.^19^ The same source was applied for the cost data and relatively similar numbers for HZ incidence were used in both models. The ZONA model included a second-dose compliance of 70%, whereas the STIKO model assumed 100%. In the STIKO model, the baseline utilities were set to 1, being in perfect health, irrespective of age, while an age-specific baseline utility value was assumed in the present model. This could explain the slightly more positive ICER results in the STIKO model, compared to the ZONA model, especially in subjects ≥ 70 YOA. One of the strengths of this model is the use of German-specific utility losses while the STIKO model reported values from Canada.^7,28^ [*submitted for publication*] We are not aware of any other German or European cost-effectiveness assessment of RZV. Recently, a comparative analysis has been performed in the US, which informed the Advisory Committee on Immunization Practices (ACIP) and reported outcomes showing cost-effectiveness in both populations ≥ 50 and ≥ 60 YOA. Based on all available evidence, ACIP made the recommendation to (1) vaccinate everyone ≥ 50 YOA, (2) revaccinate people who previously received ZVL and (3) implement RZV as preferred vaccine over ZVL.^29^

In 2014, Kawai *et al*. published a review on economic models assessing the cost-effectiveness of ZVL and made a few recommendations for future modeling, such as including the productivity loss due to HZ, the prevention of other complications than PHN, adverse events due to vaccination and most recent country-specific data on disease burden. They also highlighted the importance of assessing cost-effectiveness in smaller age cohorts.^25^ In this study, we aimed to include all those recommendations. A strength of this study is that all epidemiological, costs and utility parameters came from German sources. This analysis used the most recent data regarding disease burden and VE. At this point in time, only clinical trial data were available. Once available, it would be worth assessing the public health and economic impact of RZV on the basis of real-world effectiveness data for both the two-dose schedule and the VE for people only receiving one dose.

In conclusion, introducing a RZV universal mass vaccination in Germany is estimated to substantially reduce the burden of disease caused by HZ and PHN and to provide good value for money in the population ≥ 60 YOA.

## Methods

### Model design

For this analysis, the ZONA model was used; its structure has been described in Curran *et al*.^19^ Previously, the model only addressed questions on public health while for this analysis, components on costs and quality of life were included. Since no indirect effects can be generally expected from HZ vaccination, ZONA was designed as a static, multi-cohort Markov model built in *MS Excel* (2010). A detailed overview of the model structure is given in the supplementary material. Recurrence of HZ and subsequent development of complications were considered. A routine vaccination with RZV was compared to no vaccination, reflecting the current situation in Germany. The ZONA model followed hypothetical cohorts of 1M people over their lifetime. Outcomes of the model included costs (medical and societal) associated with the intervention, QALYs, and cases avoided. The primary outcome was the ICER in terms of costs per QALY, from the societal perspective. Both costs and outcomes are annually discounted at 3%, in the base case, following German guidelines. Differential discounting was applied in the sensitivity analyses.^30^

### Base-case analysis

In the base-case analysis, we evaluated the ICER for both populations ≥ 60 and ≥ 70 YOA. A hypothetical multi-cohort of 1M people started at 60 or 70 YOA respectively. The vaccination occurred at 60, 65 and/or 70 and 80 YOA; these cohorts were then followed over their lifetime.

### Scenario analyses

Vaccinating everyone ≥ 60 or ≥ 70 YOA would be expected to have a considerable impact on the German vaccination budget. Therefore, scenario analyses have been performed to assess the ICERs in a breakdown of the ≥ 60 YOA cohort into 60, 65 and 70 YOA assessing RZV versus no vaccination. Again, cohorts of 1M people per group of interest were followed over their lifetime. Both public health and economic impact in terms of NNV and costs per HZ and PHN cases averted were presented. These outcomes informed the discussion on the optimal age of vaccination.

### Sensitivity analyses

On the base-case analyses, two types of sensitivity analyses were performed to test the robustness of the model and its results, a DSA and a PSA. Ranges used in the sensitivity analyses are presented in the input table (Table 1). For the DSA, every base-case parameter was varied with the upper and lower bound separately and we then recorded the ICER. Results are presented in tornado diagrams. For each analysis, the PSA included 5,000 Monte-Carlo simulations using the same ranges as for the DSA. Disease-specific inputs (i.e. incidence, probabilities) and utility inputs were defined as beta distributions, costs were set to gamma distributions in the PSA and a uniform distribution was assumed for the second-dose compliance. We assumed a correlation between parameters that varied across age groups (i.e. HZ disease incidence, PHN probability, direct and indirect costs, QALY loss); therefore a correlation factor of 0.5 was applied.^31^ Results of the PSA are presented on a cost-effectiveness scatter plot and a cost-effectiveness acceptability curve. Threshold analyses were conducted on different parameters (i.e. HZ recurrence, vaccine price, vaccine efficacy (2-doses), waning of efficacy and QALY loss associated with HZ and PHN). These parameters were varied by steps of 10% to −100% and + 100% to assess the impact on the ICER and thus the potential increase or decrease of these values to reach the WTP threshold. At this time, there is no official cost per QALY threshold in Germany. As an estimate, we applied the €50,000/QALY threshold that is commonly used in other economic evaluations relevant to Germany.^32–34^

### Model inputs

The data used to populate the ZONA model were derived from published literature. We have divided the inputs into five sections; demographics, epidemiology, costs, utilities and vaccine characteristics, presented in Table 3.

**Table 3.** Input values for base-case and sensitivity analyses.

Parameter	Age group (YOA)	Base-case value	DSA (Min.)	DSA (Max.)	S.E for PSA
***Epidemiology***					
Population size**	60–64	5,202,056	N/A	N/A	N/A
	65–69	4,331,884			
	70–79	8,239,091			
	≥ 80	4,729,203			
*Source*		*DeStatis* *^35^*			
HZ – Incidence and recurrence	60–64	0.0100	0.0063	0.0103	0.0019
	65–69	0.0114	0.0072	0.0118	0.0021
	70–79	0.0134	0.0085	0.0138	0.0025
	≥ 80	0.0139	0.0094	0.0148	0.0023
*Source*		*Hillebrand et al*. *^5^*	*Schmitt-Ott et al*.*^21^*	*Hillebrand et al*. ^5^*	
PHN – Probability of HZ Cases	60–64	15.4%	5.10%	20.51%	0.053
	65–69	17.5%	5.10%	20.51%	0.063
	70–79	20.0%	6.78%	24.05%	0.067
	≥ 80	20.4%	11.17%	26.03%	0.047
*Source*		*Hillebrand et al*. *^5^*	*Horn et al*.*^22^**#*	*Horn et al*. *^22^**#*	
HZ-related fatality	60–64	0.003%	0.000%	0.013%	0.000051
	65–69	0.005%	0.002%	0.016%	0.000056
	70–74	0.010%	0.004%	0.024%	0.000071
	75–79	0.025%	0.011%	0.046%	0.000107
	80–84	0.043%	0.022%	0.076%	0.000168
	≥ 85	0.165%	0.095%	0.263%	0.000500
*Source*		*Ultsch et al*. *^10^*	*Ultsch et al*. *^10^*	*Ultsch et al*. *^10^*	
***Quality of Life***					
Baseline Utilities	60–64	0.975	N/A	N/A	
	65–69	0.976			
	70–79	0.959			
	≥ 80	0.895			
*Source*		*Paper submitted for publication*			
Disutilities HZ only	60–69	0.018	−30%	+ 30%	0.0018
	≥ 70	0.019			0.0019
*Source*		*Paper submitted for publication*	*Assumption*	*Assumption*	
Disutilities HZ and PHN	≥ 50	0.158	−30%	+ 30%	0.0161
*Source*		*Paper submitted for publication*	*Assumption*	*Assumption*	
***Costs***					
Direct medical – per HZ case	60–64	€226	€179	€270	22.45
	65–69	€226	€179	€270	22.45
	70–79	€203	€159	€252	25.00
	≥ 80	€320	€249	€394	37.76
Direct medical – per PHN case	60–64	€1,349	€714	€2,125	395.92
	65–69	€1,349	€714	€2,125	395.92
	70–79	€1,172	€717	€1,785	312.75
	≥ 80	€642	€251	€1,157	262.76
*Source direct costs*		*Ultsch et al*.*^9^*	*Ultsch et al*.*^9^*	*Ultsch et al*.*^9^*	
Indirect – per HZ case	60–64	€112	−20%	+ 20%	11.42
	65–69	€112			11.42
	70–79	€11			1.12
	≥ 80	€11			1.12
Indirect – per PHN case	60–64	€788	−20%	+ 20%	80.40
	65–69	€788			80.40
	70–79	€46			4.69
	≥ 80	€34			3.47
*Source indirect costs*		*Ultsch et al*.*^9^*	*Assumption*	*Assumption*	
***Vaccine costs***					
Price per dose	All	€110	€100	€120	
*Source*		*Assumption*	*Assumption*	*Assumption*	
Administration costs per dose	All	€7.55	€6.30	€9.43	0.959
*Source*		*See supplementary material*	*See supplementary material*	*See supplementary material*	
Adverse Events total†	60–64	€1.86	−50%	+ 100%	0.97
	65–69	€1.86			0.97
	70–79	€1.81			0.95
	≥ 80	€1.85			0.96
		*See supplementary material*	*See supplementary material*	*See supplementary material*	
***Discounting***					
Costs	All	3%	1%	5%	
Outcomes	All	3%	1%	5%	

DSA: Deterministic sensitivity analysis; HZ: Herpes zoster; PHN: Postherpetic neuralgia.

** Population size is used to define the age distribution within the 1M cohort.

* Maximum values of HZ incidence correspond to the upper bound of the 95% confidence interval (CI) reported by Hillebrand *et al*.^5^

# Horn *et al*. PHN probability estimates based on a time algorithm (minimum value) and diagnosis algorithm (maximum value), thereby covering the broadest range of probabilities.^22^

† See supplementary text for references and calculations to derive the total costs per dose.

#### Demographic inputs

The population distribution and age-related all-cause mortality rates were derived from the Federal Statistical Office (DeStatis – Statistisches Bundesamt) for the year 2015 in Germany.^35^

#### Epidemiological inputs

Several studies have estimated the HZ incidence and the frequency of associated complications in Germany.^5,9,36,37^ Hillebrand *et al*. (2015) estimated the HZ incidence and PHN probability based upon the German Pharmacoepidemiological Research Database (GePaRD) containing, for this analysis, claims data of about 7M insured people from 3 SHI, including many HZ cases (215,959 incident cases). To date, this is the most recent study from a large and well-validated database, and therefore the most accurate source for this model.^5^ In accordance with Ultsch *et al*. (2013), it was assumed that the rates of recurrent HZ episodes were the same as the rates of the first HZ occurrence, based on data from the US.^38,39^

HZ fatality figures were calculated from Ultsch *et al*. (2011), using the same approach as applied in a previous review; mortality rates reported per 100,000 person-years were calculated as the HZ case fatality rate.^10,40^

#### Costs

Direct medical and indirect costs related to HZ were taken from Ultsch *et al*. (2013), who used a retrospective database combining both patient-related billing data from a SHI and patient-related treatment documentation from a regional Association of SHI-Accredited Physicians. Individuals of all ages with a HZ diagnosis in 2005–2008 were included and observed for two years (one HZ diagnosis-free year before diagnosis and one year after HZ diagnosis).^9^

Total direct medical costs included outpatient and inpatient care, drug prescription, therapeutic appliance and sick-pay costs. For indirect costs, Ultsch *et al*. (2013) also accounted for sick-leave and co-payments.^9^ Direct medical costs for patients with HZ and HZ including PHN also include patients with other HZ-related complications. Therefore, cost of complications others than PHN were set to zero, to avoid double-counting. Indirect costs were calculated based on the costs to society minus the cost to the payer as described by Ultsch *et al*.*^9^*

A price for RZV has not been disclosed yet. Therefore, a hypothetical price of €110 per dose was assumed, reflecting the price to retailer reduced by the obligatory pharmacy and manufacturer rebates. This corresponds to a price to wholesaler of €84.5 per dose, a similar approach to other studies.^38^ We assumed vaccine administration costs of €7.55 per dose, adapted from pneumococcal vaccination in accordance with previously published literature.^22,38^

The model also included costs due to vaccination-related adverse events (See supplementary material). Hence, the total costs per dose are shown in Table 3.

#### Utility inputs

A prospective study assessing the impact of HZ and PHN on the quality of life of individuals ≥ 50 YOA in Germany was used to estimate utility inputs using the EQ-5D questionnaire. Patients were recruited when consulting primary care physicians for a first HZ episode and followed up longitudinally for a maximum of 9 months. The QALY loss associated with a HZ-only episode (i.e. no PHN) was estimated to be 0.018 in subjects 50–69 YOA and 0.019 in subjects ≥ 70 YOA, and 0.158 for a HZ episode involving PHN.^28^ [submitted for publication]

QALY losses due to adverse events were also considered. A specific source for Germany was not found, therefore similar values were applied as in a US model assessing the cost-effectiveness of ZVL.^41^

#### Vaccine characteristics

VE of RZV was evaluated in two phase III clinical trials in 16,161 subjects ≥ 50 YOA (ZOE-50) and in 14,816 subjects ≥ 70 YOA (ZOE-70), respectively.^17,18^ Details of VE estimates and waning rate calculations were given in the study presented by Curran *et al*. (2017).^19^ Based on the clinical trial data of the ZOE-50 study, it was found that there was an annual decrease of 1% per year in VE for subjects in the 50–69 YOA group. To date, data are available for 4 years post vaccination. It was assumed that VE would wane at 2.3% during the subsequent years until 70 YOA. VE was assumed to wane at 3.6% thereafter based on the ZOE-70 pooled analyses. 1-dose VE data with assumed waning were also considered as this model considers a second-dose compliance of RZV < 100%. (See supplementary material for further details).

In the model, a first-dose coverage of 40% with a second-dose compliance of 70% were assumed as done elsewhere.^19^

### Validation

Although not officially published in a peer-reviewed journal, STIKO presented a report on their website to show initial study results on cost-effectiveness for both ZVL and RZV.^20^ This report supported the recommendation not to introduce ZVL in the German population.^14^ To validate our study results, we inserted the price used by STIKO in our model and assessed the ICER.

## Trademark

Shingrix is a trademark of the GSK group of companies.

Zostavax is a trademark from Merck Sharp & Dohme Corp.

## Abbreviations

ACIP: Advisory Committee on Immunization Practices
CI: 95% confidence interval
DSA: deterministic sensitivity analysis
EMA: European Medicines Agency
GePaRD: German Pharmacoepidemiological Research Database
HZ: herpes zoster
ICER: incremental cost-effectiveness ratio
M: million
NITAG: National Immunization Technical Advisory Group
NNV: number needed to vaccinate
PHN: postherpetic neuralgia
PSA: probabilistic sensitivity analysis
QALY: quality-adjusted life year
RKI: Robert Koch Institute
RZV: Adjuvanted Recombinant Zoster Vaccine
SHI: statutory health insurance
STIKO: Ständige Impfkommission [German Standing Committee on Vaccination]
VE: vaccine efficacy
VZV: varicella-zoster virus
WTP: willingness to pay; YOA: years of age
ZONA: ZOster ecoNomic Analysis
ZVL: Zoster Vaccine Live.

## References

[1] Wiese-Posselt M, Siedler A, Mankertz A, Sauerbrei A, Hengel H, Wichmann O, Poethko-Muller C. Varicella-zoster virus seroprevalence in children and adolescents in the pre-varicella vaccine era, Germany. BMC Infect Dis. 2017;17(1):356. doi:10.1186/s12879-017-2461-2. 28525973 PMC5438501

[2] Wutzler P, Farber I, Wagenpfeil S, Bisanz H, Tischer A. Seroprevalence of varicella-zoster virus in the German population. Vaccine. 2001;20(1–2):121–124. doi:10.1016/S0264-410X(01)00276-6. 11567755

[3] Cohen JI Herpes zoster. N Engl J Med. 2013;369(18):1766–1767. doi:10.1056/NEJMc1310369. PMC4786186 24171531

[4] Pinchinat S, Cebrian-Cuenca AM, Bricout H, Johnson RW Similar herpes zoster incidence across Europe: results from a systematic literature review. BMC Infect Dis. 2013;13:.170. doi:10.1186/1471-2334-13-170. 23574765 PMC3637114

[5] Hillebrand K, Bricout H, Schulze-Rath R, Schink T, Garbe E Incidence of herpes zoster and its complications in Germany, 2005-2009. J Infect. 2015;70(2):178–186. doi:10.1016/j.jinf.2014.08.018. 25230396

[6] Opstelten W, Mauritz JW, De Wit NJ, van Wijck AJ, Stalman WA, van Essen GA Herpes zoster and postherpetic neuralgia: incidence and risk indicators using a general practice research database. Fam Pract. 2002;19(5):471–475. doi:10.1093/fampra/19.5.471. 12356697

[7] Drolet M, Brisson M, Schmader KE, Levin MJ, Johnson R, Oxman MN, Patrick D, Blanchette C, Mansi JA The impact of herpes zoster and postherpetic neuralgia on health-related quality of life: a prospective study. Can Med Assoc J. 2010;182(16):1731–1736. doi:10.1503/cmaj.091711. 20921251 PMC2972323

[8] Oster G, Harding G, Dukes E, Edelsberg J, Cleary PD Pain, medication use, and health-related quality of life in older persons with postherpetic neuralgia: results from a population-based survey. J Pain. 2005;6(6):356–363. doi:10.1016/j.jpain.2005.01.359. 15943957

[9] Ultsch B, Koster I, Reinhold T, Siedler A, Krause G, Icks A, Schubert I, Wichmann O Epidemiology and cost of herpes zoster and postherpetic neuralgia in Germany. Eur J Health Econ. 2013;14(6):1015–1026. doi:10.1007/s10198-012-0452-1. 23271349

[10] Ultsch B, Siedler A, Rieck T, Reinhold T, Krause G, Wichmann O Herpes zoster in Germany: quantifying the burden of disease. BMC Infect Dis. 2011;11:.173. doi:10.1186/1471-2334-11-173. 21679419 PMC3141411

[11] Varghese L, Standaert B, Olivieri A, Curran D The temporal impact of aging on the burden of herpes zoster. BMC Geriatr. 2017;17(1):30. doi:10.1186/s12877-017-0420-9. 28114907 PMC5259900

[12] STIKO Standard operating procedure of the German standing committee on vaccinations (STIKO) for the systematic development of vaccination recommendations, Version 3.0. Berlin; 2016 3 16th [accessed 2018 Jan 15]. https://www.rki.de/EN/Content/infections/Vaccination/methodology/SOP.pdf?__blob=publicationFile.

[13] Robert Koch Institut Standing Committee on Vaccination (STIKO). 2016 8 29 [accessed 2017 Oct 3]. http://www.rki.de/EN/Content/infections/Vaccination/Vaccination_node.html.

[14] Siedler A, Koch J, Ultsch B, Garbe E, von Kries R, Ledig T, Mertens T, Überla K, Zepp F, Hengel H Background paper to the decision not to recommend a standard vaccination with the live attenuated herpes zoster vaccine for the elderly in Germany. Bundesgesundheitsblatt. 2017;60(10):1162–1179. doi:10.1007/s00103-017-2618-6.

[15] European Medicines Agency Zostavax - EPAR - product information - summary of product chracteristics. 2018 1 18 [accessed 2018 Apr 05]. http://www.ema.europa.eu/docs/en_GB/document_library/EPAR_-_Product_Information/human/000674/WC500053462.pdf.

[16] European Commission - Public Health Community register of medicinal products for human use - Product information - Shingrix. 2018 4 03 [accessed 2018 Apr 05]. http://ec.europa.eu/health/documents/community-register/html/h1272.htm.

[17] Cunningham AL, Lal H, Kovac M, Chlibek R, Hwang SJ, Diez-Domingo J, Godeaux O, Levin MJ, McElhaney JE, Puig-Barbera J, et al Efficacy of the herpes zoster subunit vaccine in adults 70 years of age or older. N Engl J Med. 2016; 375(11):1019–1032. doi:10.1056/NEJMoa1603800. 27626517

[18] Lal H, Cunningham AL, Godeaux O, Chlibek R, Diez-Domingo J, Hwang S-J, Levin MJ, McElhaney JE, Poder A, Puig-Barberà J, et al Efficacy of an adjuvanted herpes zoster subunit vaccine in older adults. N Engl J Med. 2015;372(22):2087–2096. doi:10.1056/NEJMoa1501184. 25916341

[19] Curran D, Van Oorschot D, Varghese L, Oostvogels L, Mrkvan T, Colindres R, von Krempelhuber A, Anastassopoulou A Assessment of the potential public health impact of herpes zoster vaccination in Germany. Hum Vaccin Immunother. 2017;13(10):2213–2221. doi:10.1080/21645515.2017.1345399. 28708959 PMC5647993

[20] Ultsch B, Weidemann F, Koch J, Siedler A Projektbericht: modellierung von epidemiologischen und gesundheitsökonomischen Effekten von Impfungen zur Prävention von Herpes zoster. 2017 8 01 [accessed 2018 Apr 05]. https://www.rki.de/DE/Content/Infekt/Impfen/ImpfungenAZ/Zoster/Modellierung_Zoster_Impfung.pdf?__blob=publicationFile.

[21] Schmidt-Ott R, Schutter U, Simon J, Nautrup BP, von Krempelhuber A, Gopala K, Anastassopoulou A, Guignard A, Curran D, Matthews S, et al Incidence and costs of herpes zoster and postherpetic neuralgia in German adults aged ≥50 years: A prospective study. J Infect. 2018;76:475–482. doi:10.1016/j.jinf.2018.02.001. 29428228

[22] Horn J, Damm O, Kretzschmar M, Karch A, Siedler A, Ultsch B, Weidemann F, Greiner W, Mikolaczyk R Modellierung der effekte des varizellen-impfprogramms in Deutschland. Abschlussbericht, Version 1.2. 2014 9 16 [accessed 2017 Jun 15]. http://www.rki.de/DE/Content/Infekt/Impfen/Forschungsprojekte/abgeschlossene_Projekte/Varizellen-Impfung/Abschlussbericht.pdf?__blob=publicationFile.

[23] Bresse X, Annemans L, Preaud E, Bloch K, Duru G, Gauthier A Vaccination against herpes zoster and postherpetic neuralgia in France: a cost-effectiveness analysis. Expert Rev Pharmacoecon Outcomes Res. 2013;13(3):393–406. doi:10.1586/erp.13.19. 23537397

[24] van Hoek AJ, Gay N, Melegaro A, Opstelten W, Edmunds WJ Estimating the cost-effectiveness of vaccination against herpes zoster in England and Wales. Vaccine. 2009;27(9):1454–1467. doi:10.1016/j.vaccine.2008.12.024. 19135492

[25] Kawai K, Preaud E, Baron-Papillon F, Largeron N, Acosta CJ Cost-effectiveness of vaccination against herpes zoster and postherpetic neuralgia: a critical review. Vaccine. 2014;32(15):1645–1653. doi:10.1016/j.vaccine.2014.01.058. 24534737

[26] Prosser LA Economic evaluation of vaccination for prevention of herpes zoster and related complications. Presentation to the advisory committee on immunization practices. 2017 [accessed 2018 5] https://www.cdc.gov/vaccines/acip/meetings/downloads/slides-2017-10/zoster-03-prosser.pdf

[27] Le P, Rothberg MB Cost-effectiveness of the adjuvanted herpes zoster subunit vaccine in older adults. JAMA Intern Med. 2018;178(2):248–258. doi:10.1001/jamainternmed.2017.7431. 29297049 PMC5838796

[28] Anastassopoulou A, Curran D, Schmidt‐Ott R, Schutter U, Simon J, Poulsen Nautrup B, Matthews S Herpes Zoster and postherpetic neuralgia: quality of life and healthcare utilization, a German study; Paper presented at: ISPOR 19th Annual European Congress; 2016 10 31; Vienna (Austria). doi:10.1016/j.jval.2016.09.427

[29] Dooling KL, Guo A, Patel M, Lee GM, Moore K, Belongia EA, Harpaz R Recommendations of the advisory committee on immunization practices for use of herpes zoster vaccines. MMWR Morb Mortal Wkly Rep. 2018;67(3):103–108. doi:10.15585/mmwr.mm6703a5. 29370152 PMC5812314

[30] STIKO Modelling methods for predicting epidemiological and health economic effects of vaccinations – guidance for analyses to be presented to the German Standing Committee on Vaccination (STIKO). Berlin; last updated: 2016 3 16 [accessed 2018 Apr 05]. http://www.rki.de/EN/Content/infections/Vaccination/methodology/Guidance_for_analyses.pdf?__blob=publicationFile.

[31] Neine M, Curran D An algorithm to develop correlated multivariate non-normal (E.G. Beta, Gamma, Log-Normal) distributions to be used in probabilistic sensitivity analyses (PSAs). Value in Health. 2017;20(9):A757. doi:10.1016/j.jval.2017.08.2134.

[32] Brettschneider C, Luck T, Fleischer S, Roling G, Beutner K, Luppa M, Behrens J, Riedel-Heller SG, Konig HH Cost-utility analysis of a preventive home visit program for older adults in Germany. BMC Health Serv Res. 2015;15:.141. doi:10.1186/s12913-015-0817-0. 25884452 PMC4392621

[33] Dolk C, Eichner M, Welte R, Anastassopoulou A, Van Bellinghen LA, Poulsen Nautrup B, Van Vlaenderen I, Schmidt-Ott R, Schwehm M, Postma M Cost-utility of quadrivalent versus trivalent influenza vaccine in Germany, Using an individual-based dynamic transmission model. Pharmacoeconomics. 2016;34(12):1299–1308. doi:10.1007/s40273-016-0443-7. 27647004 PMC5110585

[34] Krejczy M, Harenberg J, Marx S, Obermann K, Frolich L, Wehling M Comparison of cost-effectiveness of anticoagulation with dabigatran, rivaroxaban and apixaban in patients with non-valvular atrial fibrillation across countries. J Thromb Thrombolysis. 2014;37(4):507–523. doi:10.1007/s11239-013-0989-6. 24221805

[35] DeStatis - Statistisches Bundesamt Bevölkerung Deutschlands bis 2060 - Ergebnisse der 13. koordinierten Bevölkerungsvorausberechnung. 2015 4 28 Wiesbaden [accessed 2017 Jun 15]. https://www.destatis.de/DE/Publikationen/Thematisch/Bevoelkerung/VorausberechnungBevoelkerung/BevoelkerungDeutschland2060_5124202159004.pdf?__blob=publicationFile.

[36] Paul E, Thiel T Zur epidemiologie der varizella-zoster-infektion. Ergebnisse einer prospektiven Erhebung im Landkreis Ansbach. [Epidemiology of varicella zoster infection. Results of a prospective study in the Ansbach area]. Hautarzt. 1996;47(8):604–609. doi:10.1007/s001050050476. 8964702

[37] Schiffner-Rohe J, Köster I, Beillat M, Lilie HM, Schubert I Ressourcenverbrauch und kosten von herpes zoster und postherpetischer neuralgie in Deutschland [Resource consumption and health care costs of Herpes zoster and postherpetic neuralgia in Germany]. Gesundh Ökon Qual Manag. 2011;16(4):216–223. doi:10.1055/s-0029-1245896.

[38] Ultsch B, Weidemann F, Reinhold T, Siedler A, Krause G, Wichmann O Health economic evaluation of vaccination strategies for the prevention of herpes zoster and postherpetic neuralgia in Germany. BMC Health Serv Res. 2013;13:.359. doi:10.1186/1472-6963-13-359. 24070414 PMC3849436

[39] Yawn BP, Saddier P, Wollan PC, St Sauver JL, Kurland MJ, Sy LS A population-based study of the incidence and complication rates of herpes zoster before zoster vaccine introduction. Mayo Clin Proc. 2007;82(11):1341–1349. doi:10.4065/82.11.1341. 17976353

[40] Bricout H, Haugh M, Olatunde O, Prieto RG Herpes zoster-associated mortality in Europe: a systematic review. BMC Public Health. 2015;15:.466. doi:10.1186/s12889-015-1753-y. 25940080 PMC4435558

[41] Le P, Rothberg MB Cost-effectiveness of herpes zoster vaccine for persons aged 50 years. Ann Intern Med. 2015;163(7):489–497. doi:10.7326/M15-0093. 26344036

