# Supplementary material for: Cost-effectiveness of the recombinant zoster vaccine in the German population aged ≥60 years old
Source: Hum Vaccin Immunother. 2018 Sep 6;15(1):34–44. doi: 10.1080/21645515.2018.1509645 (PMC6363061; doi:10.1080/21645515.2018.1509645)
Supplement: Supplemental Material [file khvi-15-01-1509645-s001.docx]

# Supplementary Material

## Model structure


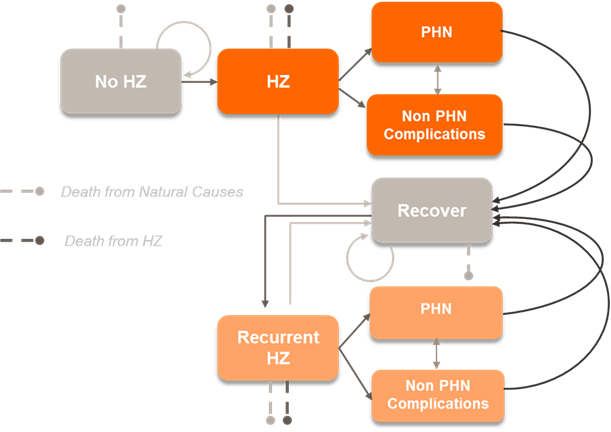


Reproduced from Curran et al. ^1^

HZ: herpes zoster; PHN: postherpetic neuralgia.

## Adverse events and associated costs

As presented elsewhere, four types of adverse events were taken into account in the ZONA model. [*submitted for publication*] Costs of local/general adverse events (AEs), which do not require medical advice were derived from Lee *et al*. (2008), being €1.12.^2^ General practitioner visit costs were calculated from ‘Physician’s Fee Schedule’ (Einheitlicher Bewertungsmaßstab; GOP 03000), assuming one visit only.^3^ Emergency room visits were calculated from ‘Physician’s Fee Schedule’, assuming an additional general practitioner visit afterwards (Einheitlicher Bewertungsmaßstab; GOP 01212 and 03000)^3^. In accordance with Lee *et al*. (2008), serious AEs were assumed to be anaphylaxis.^2^ Related to the corresponding diagnosis code (F73B), €1,812.97 were included as costs for serious adverse events.^4^ A weighted AE cost per dose is calculated by the model, based on the incidence of the four AE categories and the costs per event, per age group. This calculation has been described in more details elsewhere. [*submitted for publication*] The input table (Table 3) in this paper shows the weighted costs related to AEs per dose.

## Administration costs

In absence of vaccine administration costs defined for HZ in Germany, costs were adapted from pneumococcal vaccination, being in accordance with other authors of cost-effectiveness evaluations for HZ in Germany.^5, 6^ Regional values for GOP 89119 (as valid for the SHI AOK) were derived for 6 federal states (Berlin, Brandenburg, Hesse, Lower Saxony, North Rhine-Westphalia, Thuringia), covering approximately half of the German population. The weighted mean costs for 2017 (weighted by population size) were calculated to €7.55 (range: €6.30 – €9.43). For the second dose of RZV, the same administration costs were assumed.

## Vaccine Efficacy and Waning estimates

Vaccine efficacy (VE) estimates were taken from the two large phase III clinical trials.^7, 8^ Linear waning rates were applied as presented by Curran *et al*.^1^ The VE and waning estimates as used in this model are presented in the table below.

| **Vaccine Efficacy*** | | **Against HZ**  **% (LB-UB)** |
| --- | --- | --- |
| RZV (2 doses) | 60-69 YOA | 98.4 (95.0-100.0) |
|  | ≥70 YOA | 97.8 (94.1-100.0) |
| RZV (1 dose) | 60-69 YOA | 90.0 (58.9-98.9) |
|  | ≥70 YOA | 69.5 (24.9-89.1) |
| **Waning Rate** | | **Annual Decrease**  **% (LB-UB)** |
| RZV (2 doses) | 1-4 years after vaccination and until 69 YOA | 1.0 (0.0-2.6) |
|  | ≥5 years after vaccination and until 69 YOA | 2.3 (0.7-4.6) |
|  | ≥70 YOA | 3.6 (1.4-6.6) |
| RZV (1 dose) [Assumption] | 1-4 years after vaccination | 5.4 (1.0-7.4) |
|  | ≥5 years after vaccination | 5.1 (3.6-6.9) |

*Initial vaccine efficacy at time 0

LB: lower bound; UB: upper bound; HZ: herpes zoster; RZV: Adjuvanted Recombinant Zoster Vaccine; YOA: years of age.

1. Curran D, Van Oorschot D, Varghese L, Oostvogels L, Mrkvan T, Colindres R, von Krempelhuber A, Anastassopoulou A. Assessment of the potential public health impact of Herpes Zoster vaccination in Germany. Hum Vaccin Immunother. 2017;13(10):2213-21. doi:10.1080/21645515.2017.1345399.

2. Lee GM, Riffelmann M, Wirsing von Konig CH. Cost-effectiveness of adult pertussis vaccination in Germany. Vaccine. 2008;26(29-30):3673-9. doi:10.1016/j.vaccine.2008.04.068.

3. Kassenärztliche Bundesvereiningung. Einheitlicher Bewertungsmaßstab (EBM) Stand: 1. Quartal 2017. 2017 May 17. Berlin [accessed 2018 Apr 05]. http://www.kbv.de/media/sp/EBM_Gesamt___Stand_1._Quartal_2017.pdf.

4. Bresse X, Annemans L, Preaud E, Bloch K, Duru G, Gauthier A. Vaccination against herpes zoster and postherpetic neuralgia in France: a cost-effectiveness analysis. Expert Rev Pharmacoecon Outcomes Res. 2013;13(3):393-406. doi:10.1586/erp.13.19.

5. Horn J, Damm O, Kretzschmar M, Karch A, Siedler A, Ultsch B, Weidemann F, Greiner W, Mikolaczyk R. Modellierung der Effekte des Varizellen-Impfprogramms in Deutschland. Abschlussbericht, Version 1.2. 2014 Sep 16 [accessed 2017 Jun 15]. http://www.rki.de/DE/Content/Infekt/Impfen/Forschungsprojekte/abgeschlossene_Projekte/Varizellen-Impfung/Abschlussbericht.pdf?__blob=publicationFile.

6. Ultsch B, Weidemann F, Reinhold T, Siedler A, Krause G, Wichmann O. Health economic evaluation of vaccination strategies for the prevention of herpes zoster and postherpetic neuralgia in Germany. BMC Health Serv Res. 2013;13:359. doi:10.1186/1472-6963-13-359.

7. Cunningham AL, Lal H, Kovac M, Chlibek R, Hwang SJ, Diez-Domingo J, Godeaux O, Levin MJ, McElhaney JE, Puig-Barbera J, et al. Efficacy of the Herpes Zoster Subunit Vaccine in Adults 70 Years of Age or Older. N Engl J Med. 2016;375(11):1019-32. doi:10.1056/NEJMoa1603800.

8. Lal H, Cunningham AL, Godeaux O, Chlibek R, Diez-Domingo J, Hwang SJ, Levin MJ, McElhaney JE, Poder A, Puig-Barbera J, et al. Efficacy of an adjuvanted herpes zoster subunit vaccine in older adults. N Engl J Med. 2015;372(22):2087-96. doi:10.1056/NEJMoa1501184.
